# Flow Cytometry Detection of Sperm DNA Fragmentation and Apoptotic Markers in the Semen of Infertile Males

**DOI:** 10.1155/2021/9531775

**Published:** 2021-07-17

**Authors:** Huda Mossa Omran, Moiz Bakhiet, Volker Ehemann

**Affiliations:** ^1^Department of Molecular Medicine, College of Medicine and Medical Sciences, Princess Al-Jawhara Center for Genetics and Inherited Diseases, Arabian Gulf University, Manama, Bahrain; ^2^Pulse Health Training Center, Manama, Bahrain; ^3^Institute of Pathology, University Hospital Heidelberg, Heidelberg, Germany

## Abstract

The effect of sperm molecular defects on fertilization and pregnancy outcome after assisted reproductive therapy (ART) is widely documented by both research and clinical societies. Sperm DNA fragmentation and abnormal chromatin condensation represent critical causes of male infertility. Advanced androgenic techniques for accurately identifying molecular defects help in selecting an appropriate treatment strategy. Additionally, specific markers of apoptosis are increasingly important in predicting male infertility. The ability of flow cytometry to estimate the quantity of sperm with DNA fragmentation or damage and multifactor measurements in immotile sperm have made this developed technique essential in fertility centers. The study is aimed at assessing the level of DNA fragmentation and apoptosis by measuring flow cytometry using new techniques. Flow cytometry analysis revealed a varying degree of DNA damage. It was able to quantify the degree of impairment even in samples with minimal DNA fragmentation. DNA damage was observed even in samples that were considered normal with a routine semen analysis. Flow cytometry was sensitive to changes in sperm apoptosis. Elevated p53 activity levels were associated with high DNA fragmentation. Meanwhile, B-cell lymphoma 2 (Bcl-2) activities showed a different pattern. In conclusion, flow cytometry for sperm DNA fragmentation and markers of apoptosis can be a valuable tool in assisted reproductive centers.

## 1. Introduction

Exploring the impact of sperm DNA and apoptotic changes on male subfertility is being important along with the standard semen analysis [1–3]. Zini et al. [4] reported that sperm DNA damage comprises a predictive factor for pregnancy loss after ART. The need for a precise approach to accurately measure this factor is increased. Several techniques in the andrology lab have been used to evaluate these markers [5]. The sperm chromatin dispersion (SCD) test is a method based on the shape of the characteristic halo that is created when sperm nuclear proteins are removed by acid denaturation [6, 7]. It is a direct method but with interlaboratory variability. The comet is another technique that qualitatively measures sperm DNA damage by visualising single- and double-strand breaks using electrophoresis [5]. A double-strand DNA appears at the head of a comet. The full head and tail emerge, while damaged double- and single-strand DNA fragments move towards the tail part. Therefore, this essay is an immensely useful tool to measure the DNA fragmentation index (DFI, %), which indicates the number of cells with DNA damage [8], and high DNA stainable (HDS) (%), which indicates the proportion of the histone-to-protamine transition in the immature sperm [9]. The disadvantages of this test are that the testing requires expensive equipment and a high concentration of sperm and that the reference range for the sample needs to be calibrated [5].

Flow cytometry is a potent molecular technique that has the ability in measuring several markers accurately and in a short time. In an andrology laboratory, it can be used to differentiate between several types of DNA abnormalities, apoptosis markers, the detection of antisperm antibodies, and others [10, 11].

FCM principles depend on calculating the ratio of single- and double-stranded DNA staining of sperm nuclei with DNA fluorescent stain. During FCM, exposure to acid usually results in denaturing of double-stranded DNA in spermatozoa whose chromatin structure is altered [12, 13].

Apoptosis or programmed cell death (PCD) in male germ cells normally occurs in the prospermatogonia (gonocytes) layer in the differentiating testis during fetal life [14]. Apoptosis usually occurs during normal spermatogenesis and accounts to the loss of up to 75% of potential sperm number, where only 25% of the expected number of primary spermatocytes is produced from the spermatogonia A [15]. Impairment of apoptosis at this stage generates a male infertility phenotype, while apoptosis in the mature spermatozoa appears to be significantly involved in the production of a subfertile state [16].

Apoptotic features that detected in the PCD of germ line include the following: condensation of the chromatin, membrane leakage, endoplasmic reticulum Ca^2+^ pool depletion, release of cytochrome c from mitochondria, downstream caspase activation, generating substrate cleavage, endonuclease activation, and DNA fragmentation [17–20]. Apoptotic markers that may be detected in ejaculated spermatozoa include Bcl-xl, p53, B-cell lymphoma-2 Bcl-2, caspase-3, caspase-8, caspase-9, Fas receptors, and DNA strand breaks. However, DNA strand breaks and apoptotic markers do not always exist together [14].

The guardian of the genome or p53 is an important apoptotic marker. It is called so because of its molecular weight. It is a sequence-specific transcriptional factor that plays an important role in controlling the quality of sperm production. It is usually present in very small amounts in normal sperm [14, 20]. Genital tract infection, varicocele, and testicular cancers are commonly linked with abnormal spermatogenesis and high level of fragmented DNA and p53 [21, 22]. Omran et al. [23] also revealed that smoking could induce DNA fragmentation of human spermatozoa [23].

## 2. Materials and Methods

### 2.1. Ethics

The study was approved by the Ethics Committee of Arabian Gulf University and Bahrain Ministry of Health. All participants gave written informed consent.

### 2.2. Sample Collection

Semen samples were obtained by masturbation after at least 3 days of abstinence. The samples were subjected to analysis according to the World Health Organization criteria (2010) [24]. The samples were ejaculated into sterile containers and allowed to liquefy for at least 30 min before being processed, and all experiments were performed at least in two replicates. The study inclusion criteria were applied scheduled for a routine semen analysis as a patient at Princess Al-Jawhara fertility hospital, age 20 years and more, infertility of 1 year or more whether primary or secondary, and not currently receiving hormonal treatment. Exclusion criteria were incomplete sample and incomplete questionnaire.

### 2.3. Assessment of Semen according to the WHO Guidelines (2010)

After liquefaction, the freshly collected semen samples were assessed according to WHO criteria [24]. Seminal physical characteristics (appearance, odor, viscosity, volume, and semen pH) were evaluated. Sperm motility was assessed on the wet preparation. Sperm concentration was determined, and the presence of leukocytes and spontaneous agglutinates was noted. For sperm morphology evaluation, the slides were stained with freshly prepared Giemsa stain. Teratozoospermia index (TZI) was used to categorize the sperm morphology [25].

### 2.4. Flow Cytometry (FCM) Analysis

Cytometry analysis for sperm DNA and apoptotic markers required cell fixation with citric acid and staining with fluorescent stains (DAPI–phosphate) prior to analysis. Aliquots (100 *μ*l) of the liquefied sample were fixed with 5 ml of a citric acid solution and stored at 4°C.

For sperm DNA analysis, a 0.5 ml of the citric acid fixed cells was stained with a 3.5 ml of DAP–phosphate staining solution, mixed gently, and analyzed by FCM (8).

For apoptotic marker p53 and Bcl-2 analysis, the methods were done according to Gogolin et al. [26] and Berger et al. [27], respectively. The freshly collected samples were fixed with 80% ethanol for 24 hours prior to being analyzed by flow. 200 *μ*l of the fixed cells was filled up with 2 ml of PBS, centrifuged at 1500 rpm for 10 minutes.

The supernatant was discarded, and the rest (about 100 *μ*l) was labeled by p53-PE antibody/FITC for p53 or Mouse Anti-Human Bcl-2 for Bcl-2 analysis. Then, it was reserved in the dark for 30 minutes at room temperature. The labeled cells were counter stained with 3.5 ml of DAPI–phosphate staining solution. Finally, the stained cells were kept at room temperature in the dark for 30 minutes to be analyzed by flow cytometry. Calibrations were performed by p53 or Bcl-2 isotypes, and then, analysis was done.

SPSS 16.0 software (SPSS Inc., Chicago IL, USA) was used for statistical evaluation.

## 3. Results

Male partners of 139 infertile couples agreed to participate in the study; only eighty-four of them fulfilled the criteria for inclusion. More than half of the patients (63.1%) were in the age group of 26-35 years. Around 64.3% of patients had normal semen samples. The normozoospermic semen samples had better seminal parameters than the abnormal as shown in Table 1.

Cytometry DNA analysis had shown the different statuses of chromatin maturity and DNA integrity. The samples were classified according to Hacker-Klom et al. classification [28] into eight classes that considered only class one and two as normal. Class 4 was characterized by an increase in the percentage of diploid spermatozoa samples > 5%, whereas Class 5 contained samples' histograms with skewing of the 1 CC peak to the left.

**Figure 1:**
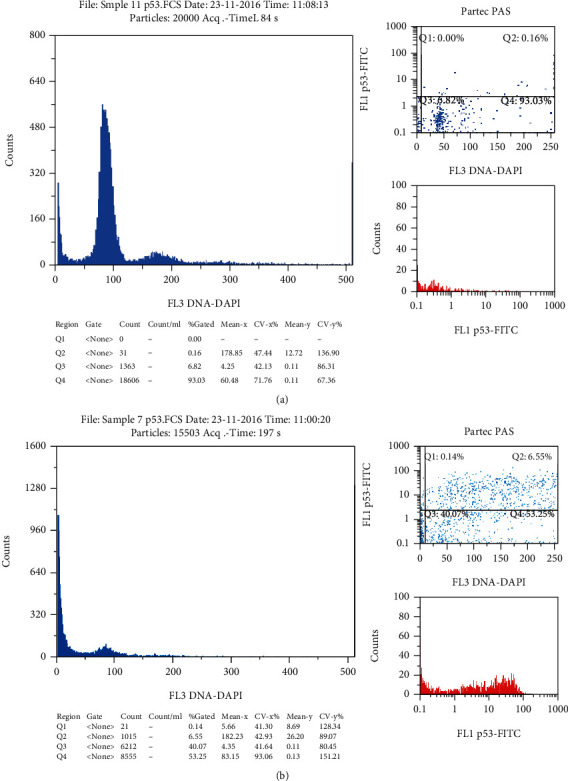
Cytometry result of p53 labeled samples: (a) normal sample with 0.16% of positively labeled cells; (b) abnormal sample with 6.55% of positively labeled cells.

**Figure 2:**
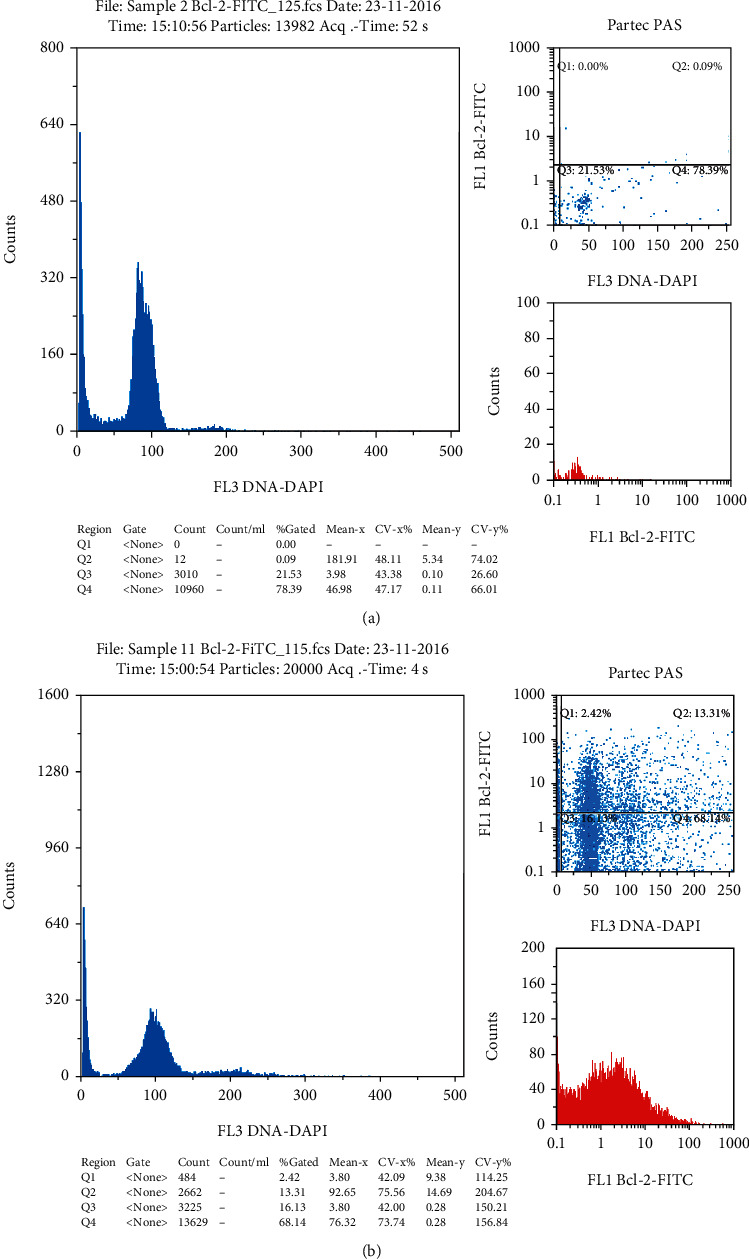
Cytometry result of Bcl-2-FITC labeled samples: (a) normal sample with 0.9% of positively labeled cells; (b) abnormal sample with 13.3% of positively labeled cells.

DNA analysis was not normal in all normozoospermic samples. About 54.8% of the semen samples demonstrated abnormal chromatin status. Samples with normal class one chromatin condensation were represented only 32.1% of the study population. Aneuploidy cells were detected in three samples with an abnormal semen analysis. Sample with abnormal p53 activity had a high level of positively labeled cells with anti-p53 antibodies (≥5%) (Figure 1). Also, samples with abnormal Bcl-2 activity had ≥5% of positively labeled cells (Figure 2).

**Figure 3:**
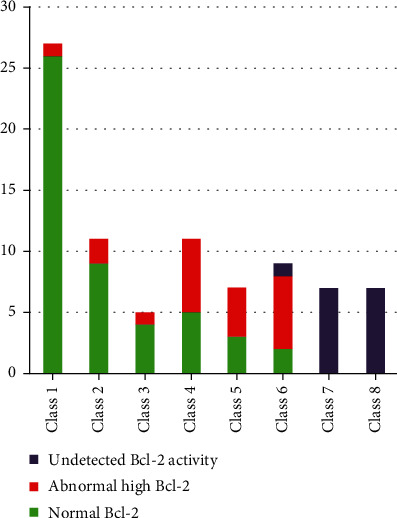
Distributions of patients according to cytometry classes vs. Bcl-2 activity.

Apoptotic markers' analysis reveals that contrary to expectation, normal chromatin condensation did not reflect standard cell cycle analysis. About 65.5% of the semen samples had an abnormally high level of positively labeled cells with FITC Mouse Anti-Human Bcl-2; some of them had normal chromatin condensation (Figure 3), while 51.2% of the semen samples had an abnormally high level of positively labeled cells with a p53-PE antibody.

**Figure 4:**
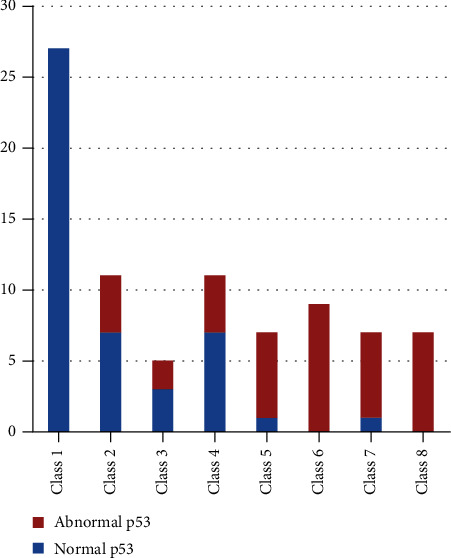
Distributions of patients according to cytometry classes vs. p53 activity.

**Figure 5:**
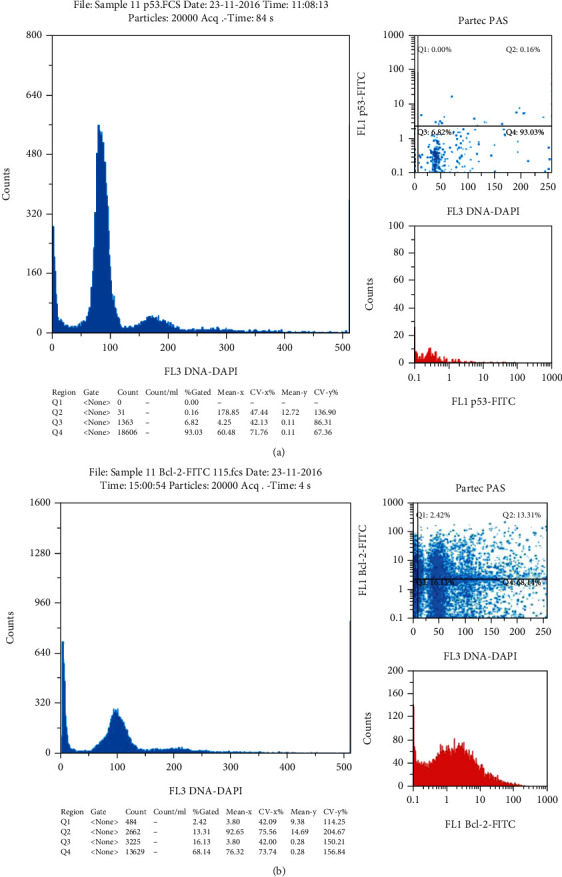
Cytometry result of sample 11: (a) normal chromatin condensation sample with normal p53 activity (0.16% of positively labeled cells); (b) abnormal Bcl-2 activity of the same sample with 13.3% of positively labeled cells.

The Bcl-2 protein activity was higher in Classes 4 and 5 than in other classes; however, no Bcl-2 activity was detected in Classes 7 and 8 (Figure 3). Comparably, Classes 7 and 8 had elevated measures of p53 activity (Figure 4). Moreover, Bcl-2 activity was even high in samples with normal chromatin condensation and normal p53 activity (Figure 5).

## 4. Discussion

Despite the widespread decline in human fertility, science has not established perfect methods for diagnosis. To date, all the IVF techniques rely in their final sperm selection on its gross morphology, not the nuclear content or DNA normality. Besides, most of the urological procedures rely principally on traditional semen analysis to assess treatment success.

The study demonstrated considerable differences in the results of the traditional analysis in comparison to those of the molecular procedures. Research showed normality of 64.3% of the study group by the routine semen analysis, whereas this figure dropped to only 34.5% after the application of the molecular methods.

The cytometry analysis of DNA integrity revealed considerable damage in sperm DNA even within the grossly normal samples. These findings indicate that selection of sperm for IVF and ICSE on the bases of gross morphology and routine analysis is not sensitive.

Because of their role in sperm maturity, analysis of apoptotic markers was performed. The p53 family of genes has an established task in the regulation of male and female germ-line production of gametes, especially in stress response. The p53 family protects against developmental defects in the progeny. Also, there is abundant evidence that p53 protein plays a critical role in female reproduction and embryonic implantation [29]. The proper response to stress signals through the adequate control of these metabolic pathways is a central element of development. Ordinarily, p53 is present in low quantity in spermatozoa; therefore, its presence in large amounts may reflect stress conditions such as cancer, oxidative stress, or testicular disease [30, 31].

The present study had the importance in measuring p53 level in human sperm population by using flow cytometry. It revealed that around 51.2% of the study population had abnormal measures of p53 protein with a significant correlation between the degree of DNA damage and p53 level (Figure 3). These findings agreed with that of Raimondo et al. [31] who found a positive correlation between the corrected p53 values measured by ELISA and the degree of DNA damage in sperm cells.

Bcl-2 was another apoptotic marker assessed in this study. Bcl-2 is expressed predominantly during sperm development. It works as an apoptotic regulator (a protooncogene) that is inserted into the outer mitochondrial, nuclear, and endoplasmic reticulum membranes with the bulk of the protein facing the cytosol [32].

Stress stimulates cytoplasmic proapoptotic proteins that move to the mitochondrial level, where antiapoptotic proteins also exist ([33], 30). The communication between proapoptotic and antiapoptotic proteins interrupts the antiapoptotic Bcl-2 function leading to the formation of mitochondrial pores and proapoptotic molecule release from the intermembrane space [33] (31). By binding to Apaf-1, these elements activate caspase-9 and through targeting caspase effectors lead eventually to cell death [34]. The Bcl-2 family enhances the cell viability, and low level of Bcl-2 harms cell survival and differentiation [35].

Around 65.5% of the study population had abnormal levels of Bcl-2. These levels were detected even in samples with normal chromatin condensation (Figure 5). The Bcl-2 protein activity was considerably high in Classes 4 and 5. However, no Bcl-2 activity was detected in Classes 7 and 8 (Figure 3). Classes 4 and 5 are considered an intermediate stage of chromatin condensation defects [28]. The increase in the Bcl-2 activity in those types of DNA defects may reflect their vital role in protection against apoptosis by interference with the mitochondrial apoptotic pathway [36], while its activity in the sample with normal chromatin condensation may indicate that sperm mitochondrial defect can exist without the existence of nuclear DNA damage and could be the cause of male infertility [37]. The absence of this activity in more severe types of chromatin defect may reflect the failure of the defense mechanism and the failure of sperm production.

The technique used in this study had many advantages including accuracy and the speed of measurements, and it can simultaneously measure the sperm chromatin condensation and aneuploidy rate. Still, a significant limitation of this technique is the expensive instrumentation and the requirement of skilled people to analyze the samples [11].

## 5. Conclusion

We have many gaps in our knowledge regarding male infertility, partly because of the restricted number of researches in our area in this crucial field and partly because of the limited value of traditional semen analysis in the assessment of infertile males. The study emphasized that future studies should apply the molecular markers in male infertility diagnosis, primarily for those involved in ART cycles. This could have a positive impact on ART results, especially if we correlate the outcome of these procedures with the findings of these assays.

## Figures and Tables

**Table 1:** Values of standard parameters of normal and abnormal samples by WHO^∗^ analysis.

Parameter	Normal (mean ± 2SD)	Abnormal (mean ± 2SD)
Concentration (×10^6^/ml)	83.18 ± 9.93	23.73 ± 5.34
Motility (% grade a)	6 0.18 ± 1.96	33.70 ± 3.96
Vitality (% alive)	68.37.84 ± 2.42	47.73 ± 4.56
Morphology (% normal form)	9.83 ± 0.68	3.46 ± 0.70
Leukocyte count (×10^6^/ml)	0.85 ± 0.15	1.23 ± 0.27

WHO: World Health Organization.

## Data Availability

The data that support the findings of this study has a sort of identifier of individual participants and researchers reserved to send it.
